# Orthosis Effects on the Gait of a Child with Infantile Tibia Vara

**DOI:** 10.1155/2015/406359

**Published:** 2015-05-21

**Authors:** Serap Alsancak, Senem Guner

**Affiliations:** Department of Orthopedic Prosthetics and Orthotics, Vocational School of Health Services, Ankara University, Fatih Street 197/A Gazino, Kecioren, 06280 Ankara, Turkey

## Abstract

Infantile tibia vara (ITV) is an acquired form of tibial deformity associated with tibial varus and internal torsion. As there is currently insufficient data available on the effects of orthotics on gait parameters, this study aimed to document the influence of orthosis on walking. A male infant with bilateral tibia vara used orthoses for five months. Gait evaluations were performed pre- and posttreatment for both legs. The kinematic parameters were collected by using a motion analysis system. The orthotic design principle was used to correct the femur and tibia. Posttreatment gait parameters were improved compared to pretreatment parameters. After 5 months, there was remarkable change in the stance-phase degrees of frontal plane hip joint abduction and knee joint varus. We found that orthoses were an effective treatment for the infantile tibia vara gait characteristics in this patient. Full-time use of single, upright knee-ankle-foot orthosis with a drop lock knee joint and application of corrective forces at five points along the full length of the limb were effective.

## 1. Introduction

Studies on surgical correction, orthotic applications, and spontaneous healing of infantile tibia vara (ITV) have been performed since the 1960s [1–9]. Most of them were radiographic evaluations describing metaphyseal-diaphyseal proximal tibial angles and the progression of distal femoral deformity [2, 10, 11]. Body weight and early walking were found to be factors that contributed to the development of the condition, which is not seen in nonambulatory patients. Ligamentous laxity and the lateral thrust of the knee in the stance phase of gait have been cited as determining factors [1, 12] and abnormally increased knee internal rotation and hip external rotation moments have been observed [13].

Nonsurgical treatment of ITV has been somewhat controversial. However, recent studies have reported success when knee-ankle-foot orthosis (KAFO) was used nearly full-time to improve biomechanics in patients under 4 years of age [14–16]. The effectiveness of orthoses with corrective forces and/or distraction systems is related to their ability to relieve weight bearing stresses on the medial physeal region of the proximal tibia. Alsancak et al. reported that a single upright KAFO with a drop lock knee joint was effective for the treatment of ITV in 22 children of about 31 months of age [14]. The duration of treatment in that study was nearly 6 months. A case report by Whiteside of two children with an approximate age of 46 months showed that double solid upright KAFOs with free knee motion significantly improved ITV after 17 months of treatment [15]. Orthotic management of ITV with drop lock or unlocked knee joints, single or double upright KAFOs applying four or five corrective forces or a distraction system or variations of these devices have been reported. However, none of the studies evaluated patient gait parameters after orthotic treatment.

The aim of this report was to evaluate the effect of treatment with a single upright KAFO with a drop lock knee joint on hip and knee walking kinematics in an ITV patient.

## 2. Case Description and Methods

A male ITV patient with an age of 2.5 years, a weight of 14 kg, and a height of 90 cm was referred to the clinic at our university by an orthopedic surgeon. The radiographic classification of the patient was Langenskiöld Stage II, and the deformity was judged not to be due to traumatic, congenital, metabolic, or infectious causes. The study was approved by the Ethics Committee of Ankara University.

**Figure 1:**
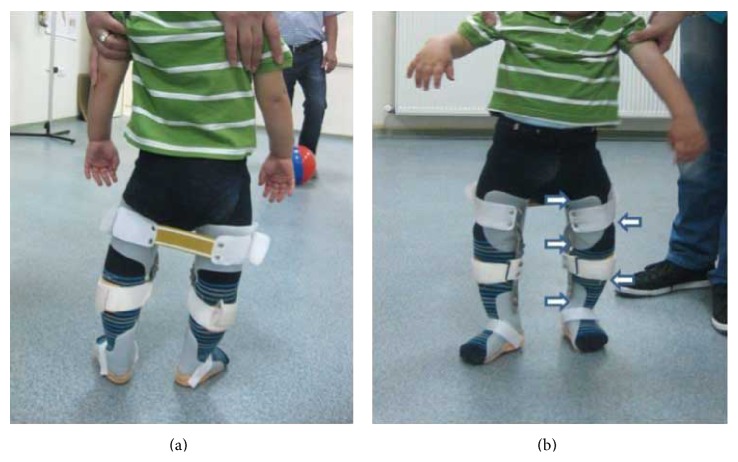
Anterior and posterior view of KAFOs application with flexible posterior strap (a) and mediolateral forces (b).

**Figure 2:**
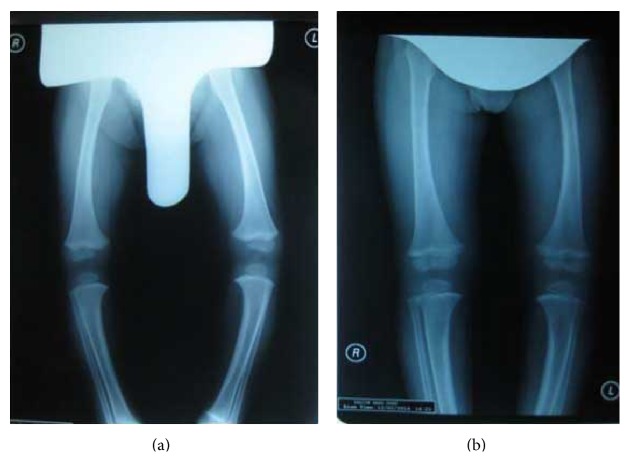
Anteroposterior radiographs of lower extremity: (a) pretreatment, (b) posttreatment.

The child was fitted with bilateral single medial metal upright KAFOs with drop lock knee joints. The orthoses applied five constant corrective forces to the femur and proximal tibia, and the lateral corrective band applied across the tibia produced a rigid column (Figure 1) [14]. The orthosis was used full-time until the first follow-up visit at 3 months after application. The orthosis was removed for 3 hours every day from beginning of treatment and evaluation after 3 months. The child was subjected to clinical examinations and radiologic evaluations before treatment and at 3- and 5-month follow-up visits (Figure 2). Stretching exercises directed at the tensor fascia lata and strengthening exercises for the knee flexor and extensors and abdominal and back muscles were taught to the families. A classic massage for the lower extremities was also taught to the families during this process.

Three dimensional gait analysis was conducted in a gait laboratory using a Vicon motion analysis system (Vicon Nexus, Oxford Metrics, Oxford, UK) with six infrared cameras at 240 Hz. Before data collection, each camera and force plate was calibrated. Data was collected after several practice trials. The average of five trials for each barefoot walking condition was calculated. The patient walked a distance of 10 m at a self-selected speed in the gait laboratory before treatment and at each follow-up visit. We used Helen Hayes marker protocol in gait analysis.

## 3. Findings and Discussion

**Figure 3:**
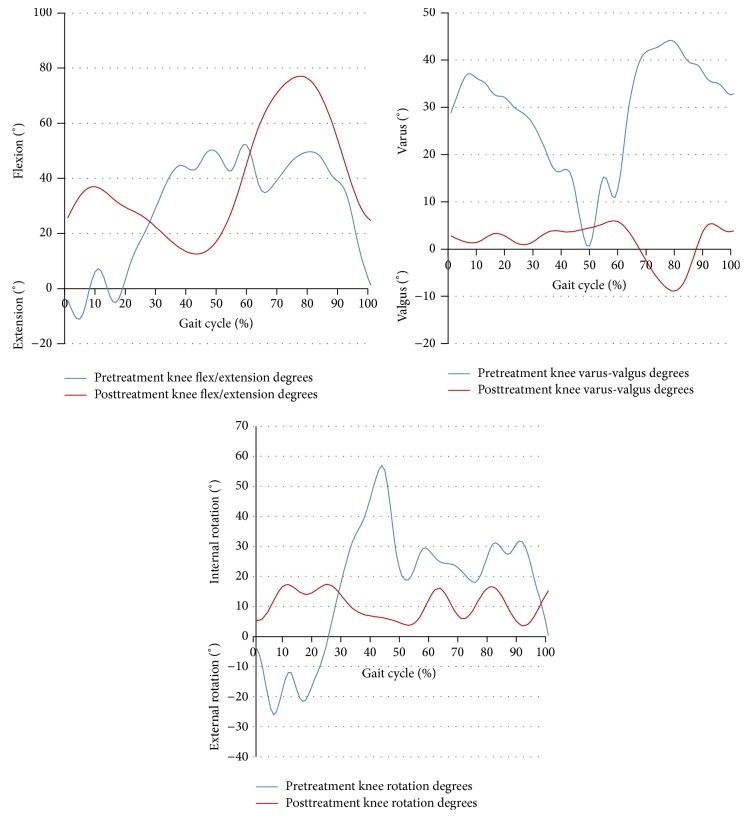
Knee joint kinematic degrees.

**Figure 4:**
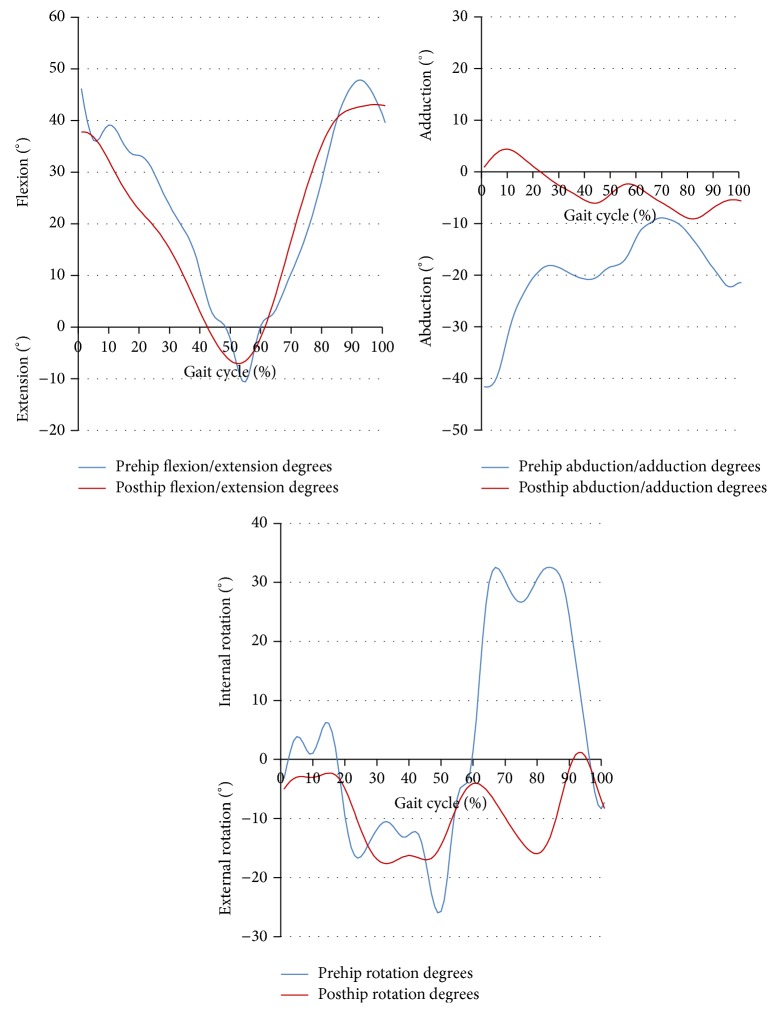
Hip joint kinematic degrees.

The results of the kinematic analysis of the hip and knee joint walking parameters are shown in Tables 1 and 2. The mean pre- and posttreatment values of flexion-extension excursion of the hip and knee joints were different. Before treatment, the lower extremity kinematic assessments indicated knee hyperextension and insufficient knee flexion during the early stance phase, with external knee rotation, knee flexion, and increased internal rotation during the late stance phase. Knee varus degree and degrees of hip flexion-extension and abduction were increased during both the early and late stance phases. After 5 months of orthotic treatment, the knee-flexion wave occurring in the early stance phase and knee extension during the late stance phase had increased, and knee flexion was closer to the normal curve. By 2 years of age, we observed a more clearly defined knee-flexion wave, an increased hip adduction in stance, and a decreased external rotation of the hip [17]. The primary change in the knee-flexion-extension curve by age showed gradual development of an initial knee-flexion wave. It should be noted that the term initial knee-flexion wave describes the flexion of the knee during a loading response and a subsequent extension during midstance. The abduction-adduction excursion of the hip and varus/valgus excursion of knee joint decreased significantly with treatment. Degrees of knee varus and hip abduction decreased significantly. Knee varus decreased nearly 30° during the early stance phases and 20° during the late stance phases (Figures 3 and 4). The excursion of the hip and knee joint rotation degrees in the sagittal plane observed after treatment were significantly different from those observed before treatment. Degrees of knee internal rotation and hip external rotation declined during late stance. Kinematic joint angles were within the closed normal curve for a child of 2 years of age. We think that the flexible posterior strap had a positive impact on the degrees of hip and knee external rotation in the horizontal plane. Application of the single upright KAFO with drop lock knees did not have any adverse effects on knee motion in the sagittal plane at the end of treatment.

**Figure 5:**
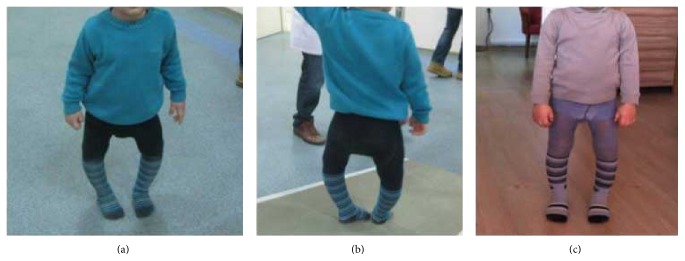
Before treatment (a, b) and after treatment (c).

To our knowledge there have been no studies reporting the influence of orthotic treatment on gait performance in ITV patients. The results of this evaluation showed significant improvement in the kinematic gait pattern of the hip and knee joints after KAFO treatment (Figures 2 and 5).

Most specialist evaluations indicate that a mature gait is present in normal children by age 5. However, in an evaluation of 309 normal children, Sutherland concluded that a mature gait pattern is established in most children by age 4 [18]. Hillman et al. reported temporal and distance parameters in normal children that supported a normal walk ratio and stride length as an idiosyncratic feature of gait from the age of 7–11 years [19]. Many authors believe that treatment by orthosis is effective in the early stages of ITV [20–28]. Our study showed successful orthotic treatment at 2.5 years of age. Alsancak et al. advised that KAFO was effective in children between 1.5 and 3.5 years of age. Bilateral orthotic treatment duration is longer than unilateral treatment of patients with ITV. Blount advised that his bowleg brace was used at night in patients younger than 2 years of age [29]. Loder and Johnston reported successful outcomes in 12 of 23 extremities in patients with Stage I-II disease, but the success rate was only 50% with orthotic treatment [30]. They concluded that orthoses were indicated only for children between 1.5 and 2.5 years of age. Full-time use of the orthosis at the beginning of treatment was important in our study.

Improved gait parameters revealed that use of a KAFO was a very effective treatment for ITV in this child. Future research should incorporate larger patient and control groups to further evaluate the effectiveness of orthosis treatment.

## Figures and Tables

**Table 1:** The kinematics of the hip and knee joints total excursion degree during pre- and posttreatment in ITV.

Parameters	Knee joint			Hip joint		
	Flexion-extension (°)	Abduction-adduction (°)	Rotation (°)	Flexion-extension (°)	Abduction-adduction (°)	Rotation (°)
Pretreatment	63 ± 33	44 ± 75	82 ± 80	58 ± 40	50 ± 60	58 ± 53
Posttreatment	65 ± 25	14 ± 84	20 ± 96	50 ± 11	13 ± 52	18 ± 83

**Table 2:** The kinematics of the hip and knee joints maximum degree during pre- and posttreatment in ITV.

Parameters	Knee joint			Hip joint		
	Max knee flexion (°)	Max knee abduction (°)	Max knee internal rotation (°)	Max hip flexion (°)	Max hip abduction (°)	Max hip external rotation (°)
Pretreatment	52.22	44.14	56.78	47.83	−41.68	−25.89
Posttreatment	77.04	5.97	17.36	43.05	−9.14	−17.64
